# Variation in pain related to systemic lupus erythematosus (SLE): a 7-year follow-up study

**DOI:** 10.1007/s10067-018-4079-1

**Published:** 2018-04-14

**Authors:** Eva Waldheim, Sofia Ajeganova, Stefan Bergman, Johan Frostegård, Elisabet Welin

**Affiliations:** 1 Division of Nursing, Department of Neurobiology, Care Science and Society, Karolinska Institutet, Stockholm, Sweden; 2 Department of Medicine, Huddinge H7, Unit for Gastroenterology and Rheumatology, Karolinska Institutet, Stockholm, Sweden; 3 Primary Health Care Unit, Department of Public Health and Community Medicine, Institute of Medicine, The Sahlgrenska Academy, University of Gothenburg, Gothenburg, Sweden; 4 Spenshult Research and Development Center, Halmstad, Sweden; 5 Department of Clinical Sciences, Section of Rheumatology, Lund University, Lund, Sweden; 6 Unit of Immunology and Chronic Disease, Institute of Environmental Medicine, Karolinska Institutet, Stockholm, Sweden; 7 Department of Medical and Health Sciences, Linköping University, Linköping, Sweden; 8 Department of Neurobiology, Care Sciences and Society, Karolinska Institutet, Stockholm, Sweden

**Keywords:** Anxiety, Depression, Fatigue, Health-related quality of life, Pain, SLE

## Abstract

We have previously shown that most patients with systemic lupus erythematosus (SLE) reported low degree of SLE-related pain. However, 24% of the patients reported high degree of SLE-related pain, more fatigue, anxiety and depression, and worse health-related quality of life (HRQoL). To explore SLE-related pain, the presence of long-standing widespread pain, and patient-reported outcomes (PROs) after 7 years. Sixty-four out of 84 patients participated in a 7-year follow-up of the original survey and completed the same questionnaires answered at inclusion: pain (VAS 100 mm), fatigue (MAF), HRQoL (SF-36), anxiety and depression (HADS), and, if appropriate, a pain-drawing. Differences between inclusion and follow-up (change) were calculated. The patients with a low degree of SLE-related pain at inclusion reported no changes at follow-up in pain and PROs except for worsening in physical function in SF-36, median change (IQR) 0 (− 10 to 5), *p* = 0.024. Half of the patients with high degree of pain at inclusion reported decreased pain at follow-up, median change (IQR) 45 (35 to 65), *p* = 0.021; fatigue, 8 (8 to 17), *p* = 0.018; anxiety, 4 (1 to 4), *p* = 0.035; and depression, 4 (2 to 5), *p* = 0.018 and improvements in most dimensions of SF-36. The remaining half of the patients reported no changes regarding pain and PROs except for a worsening in vitality in SF-36, 20 (15 to 35), *p* = 0.0018. All patients with remaining high level of pain indicated long-standing widespread pain. After 7 years, a subgroup of patients with SLE reported remaining high level of SLE-related pain and a high symptom burden, including long-standing widespread pain. Such patients require more observant attention to receive appropriate treatment.

## Introduction

### Systemic lupus erythematosus

Living with the chronic disease systemic lupus erythematosus (SLE) may imply many difficulties. Beyond organ-specific symptoms and unpredictable courses [1], patients are often reported to suffer from pain and fatigue [2, 3] and also worsened health-related quality of life (HRQoL) [4–6]. It has been also reported that patients with SLE have impaired mental health [7–9] where depression and cognitive dysfunction are the most common psychiatric manifestations [8]. No cure is currently available, but in recent decades, new treatment opinions and strategies have been developed, leading to better disease control and relief of symptoms [10].

### Pain in SLE

Pain is a frequent self-reported symptom in SLE [2, 5] and is often one of the first symptoms of the disease [3, 11]. Commonly, pain in SLE is musculoskeletal [3], but other frequently described types of pain are headache, abdominal pain, and pain caused by Raynaud’s phenomenon [12]. Pain in SLE has been described as obtrusive and unpredictable, sometimes of a continuous nature but also with rapid changes in intensity and location [1]. Pain has also been reported to be a symptom which health professionals do not pay sufficient attention to [13–15]. Although pain is a commonly reported symptom in SLE, there is a large variation between different patients [16].

We have previously shown that nearly one quarter of a cohort with patients with SLE reported a high degree of pain on a visual analogue scale (VAS) ≥ 40 mm, and these patients also reported more fatigue, reduced health-related quality of life, and more signs of anxiety and depression [16, 17].

Considering the chronic nature of SLE and the great symptom burden that disease-related pain and associated symptoms can mean for the patients, it seems important to investigate the long-term course of SLE-related pain.

The aim of the present study was to explore self-reported SLE-related pain after 7 years in the previously studied cohort, as well as fatigue, health-related quality of life, and mental health. In addition, characteristics and disease activity were captured.

## Participants and methods

### Study population

The studied cohort comprised 84 patients with SLE who were recruited consecutively in the period from 2006 to 2008 from one rheumatology centre. Also, 91 age- and sex-matched population controls were recruited. Characteristics of the cohort and the recruitment process are described in our previous studies [16–18].

In the inclusion study, the average of SLE-related pain intensity that patients experienced in the last week was measured using VAS 100 mm. The distribution of reported pain identified a clear cutoff value of 40 mm on the VAS [16]. The patients who reported SLE-related pain < 40 mm on VAS were classified as the low pain group (*n* = 64), and those who reported pain ≥ 40 mm on VAS were classified as the high pain group in our study (*n* = 20).

Data for the present 7-year follow-up study was collected between 2013 and 2015. In all, it was possible to follow 64 out of 84 patients and 68 out of 91 controls from the original inclusion study. Reasons for not participating in the follow-up study for the patients were death (*n* = 4), migration (*n* = 3), unable to participate due to illness (*n* = 2), and unwilling to participate (*n* = 11) and for the controls: migration (*n* = 1), unable to participate due to illness (*n* = 5) and unwilling to participate (*n* = 17). There was no significant difference in age (years) between those patients who participated median (IQR) 52 (38.5 to 64) and those who did not 54.3 (45 to 64.6) (*p* = 0.289 for between group difference) and those controls who participated 56 (46.5 to 66) and those who did not 49.7 (38.2 to 67.5) (*p* = 0.146), and there was no significant difference in disease duration (years) among the patients who participated 16 (11.5 to 22.5) and who did not participate 16 (13 to 25) (*p* = 0.74) in this follow-up study.

**Fig. 1:**
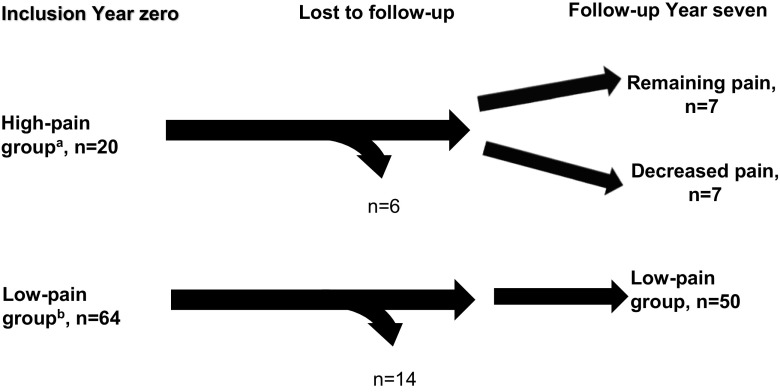
Chart of participating patients with SLE as grouped by self-reported SLE-related pain. ^a^Patients who reported SLE-related VAS pain score ≥ 40 mm at inclusion (year 0). ^b^Patients who reported SLE-related VAS pain score < 40 mm at inclusion (year 0)

For the purpose of this study, the patient groups defined by SLE-related pain at inclusion (year 0) were asked to complete the questionnaires at the follow-up assessment by a rheumatologist and a research nurse (year 7) (Fig. 1).

### Disease activity and damage

Disease activity was measured with the Systemic Lupus Activity Measure (SLAM) [19] and the Systemic Lupus Erythematosus Disease Activity Index (SLEDAI) [19] (without the laboratory parameters: lymphocytes, complement, and ds-DNA). SLAM measures disease activity during the last month and SLEDAI within the last 10 days. Disease damage was measured by the Systemic Lupus International Collaborating Clinics, American College of Rheumatology (SLICC/ACR damage index) [19]. The patient’s and the rheumatologist’s perceptions of disease activity were measured by a VAS (100 mm) which was included in the SLAM measurement [19].

### Self-assessment questionnaires

Pain intensity was measured by VAS (100 mm) [20, 21] and was connected to the questions “How much pain have you experienced on average over the previous week?” and “How much SLE-related pain have you experienced on average over the previous week?” VAS (100 mm) was also connected to the question “To what extent has SLE-related pain been a problem for you over the previous week?”

To measure the clinical pain quantitatively, the total index and numbers of pain descriptors in the short form of the McGill Pain Questionnaire (SF-MPQ) [22] were used. The total index range was 0–45, with higher values indicating more pain.

The distribution of pain was captured by a pain drawing with predefined body regions [23].

The global fatigue index (GFI) of the multidimensional assessment of fatigue (MAF) [24–26] was used to measure fatigue. Ranges of MAF/GFI were from 1 to 50, with higher values indicating more severe fatigue.

The Hospital Anxiety and Depression Scale (HADS) [27, 28] was used to measure symptoms of anxiety and depression. Scores were summarised in a total index for anxiety and a total index for depression. A total index of seven or less indicated no symptoms, scores of 8–10 indicated mild to moderate symptoms, and a score of 11 or more indicated significant symptoms of anxiety or depression.

The Medical Outcomes Survey-Short Form 36 (SF-36), standard Swedish version 1.0, was used to measure health-related quality of life (HRQoL) [29, 30]. This questionnaire measures physical and mental health using questions categorised into eight dimensions. The scores range from 0 to 100 and a higher score indicates better health.

### Statistics

The change between year 0 and year 7 in VAS pain and index for fatigue, anxiety, and depression were measured by calculating the change between the variables at year 0 and at year 7 (year 0 minus year 7). For all variables, the change represented an improvement if it was positive, and a worsening if negative. The change for health-related quality of life (SF-36) was calculated as a measure at year 7 minus year 0 because a higher value of SF-36 means better health-related quality of life. Descriptive results are presented as median and interquartile range (IQR) and nonparametric statistical tests were used for comparative analysis due to the non-normally distributed data and different sizes of the groups compared. A *p* value less than 0.05 was considered statistically significant.

The statistical analyses were conducted using the software STATISTICA 12 (Stat Soft Scandinavia AB, Uppsala, Sweden).

### Ethics

The study was approved by the Stockholm Regional Ethical Review Board and all participants gave their written informed consent.

## Results

Before characterisation of the participants, sub-grouping was performed based on reported SLE-related pain (Fig. 1).

In the whole patient group (*n* = 64), no statistical differences were found between year 0 and year 7 in respect to reported overall pain and SLE-related pain (Table 1).

**Table 1:** Pain in patients with SLE grouped by self-reported SLE-related pain and in controls at year 0 and year 7

	All patients, *n* = 64				Patients with low pain, *n* = 50 (78%)			Patients with decreased pain, *n* = 7 (11%)			Patients with remaining pain, *n* = 7 (11%)			Controls, *n* = 68		
	Year 0		Year 7	*p* ^a^	Year 0	Year 7	*p* ^a^	Year 0	Year 7	*p* ^a^	Year 0	Year 7	*p* ^a^	Year 0	Year 7	*p* ^a^
Overall pain, VAS, mm^b^	17 (3 to 45)		23 (6 to 45)	0.98	13 (3 to 23)	18 (4 to 35)	0.21	67 (51 to 71)	14 (10 to 44)	0.031*	78 (46 to 96)	64 (52 to 75)	0.92	5 (0 to 29)	11 (2 to 30)	0.09
Change^c^ in overall pain, mm^b^	–		0 (− 12 to 15)	–	–	− 1 (− 12 to 6)	–	–	48 (31 to 61)	–	–	− 0.5 (− 18 to 35)	–	–	− 2(− 13 to 14)	–
SLE-related pain, VAS, mm^b^	11 (2 to 31)		11 (1 to 33)	0.92	7 (1 to 16)	8 (1 to 22)	0.19	70 (60 to 72)	15 (2 to 37)	0.021*	67 (47 to 83)	66 (55 to 73)	0.87	–	–	–
Change^c^ in SLE-related pain, mm^b^	–		1 (− 7 to 7)	–	–	0 (− 6 to 2)	–	–	45 (35 to 65)	–	–	− 13(− 20 to 28)	–	–	–	–
Problem SLE-related pain, VAS, mm^b^	–		12 (2 to 32)	–	–	6 (1 to 23)	–	–	15 (3 to 29)	–	–	54 (47 to 88)	–	–	–	–
Remaining pain > 3 months^d^	–		36 (56)	–	–	25 (50)	–	–	4 (57)	–	–	7 (100)	–	–	34 (50)	–
Chronic widespread pain/ACR90 [31]^d^	–		20 (31)	–	–	13 (26)	–	–	0 (0)	–	–	7 (100)	–	–	9 (13)	–
Number of body regions with pain > three months^b^	–		7 (4 to 11)	–	–	6 (4 to 9)	–	–	4 (3 to 4)	–	–	11 (9 to 15)	–	–	3 (2 to 4)	–
Use of analgesics, regular^d^	–		–	–	–	12 (24)	–	–	1 (14)	–	–	6 (86)	–	–	6 (7)	–
Use of analgesics, as needed^d^	–		–	–	–	18 (36)	–	–	4 (57)	–	–	1 (14)	–	–	41 (48)	–
No use of analgesics^d^	–		–	–	–	20 (40)	–	–	2 (29)	–	–	0 (0)	–	–	39 (45)	–

*p* value defines differences between inclusion (year 0) and follow-up (year 7), – not applicable or not assessed at year 0

^a^Wilcoxon matched pairs test

^b^Medians with IQR (interquartile range)

^c^Change between year 0 and year 7

^d^Numbers (%)

*Statistically significant value

When dividing the patients into the groups by pain intensity at inclusion, the low pain group (*n* = 50) reported statistically similar levels of overall pain as well as SLE-related pain in the last week at year 0 and at year 7 (Table 1). However, the high pain group (*n* = 14) reported lower levels of overall pain at year 7, VAS median (IQR) 49 (14 to 70) compared to year 0, 70 (49 to 79) (*p* = 0.050) as well as SLE-related pain at year 7, VAS median 43 (15 to 66) and 69 (50 to 72) (*p* = 0.035), respectively. The change of SLE-related pain at year 7 was greater in the high pain group, 32 (− 13 to 48), while it was 0 (− 6 to 2) in the low pain group.

Half of the patients in the high pain group (*n* = 7) reported a lower level of SLE-related pain at year 7 and the other half of the patients in the high pain group (*n* = 7) reported similar levels of SLE-related pain at year 0 and at year 7 (Table 1).

All the patients with remaining pain (*n* = 7) reported pain lasting more than 3 months and the pain drawing indicated chronic widespread pain according to the definition by Wolf [31] (Table 1).

There was no significant difference in overall pain between the controls and the patients with low pain and those with decreased pain (Table 1) at year 7. Compared to the whole patient group as well as to the patients with remaining pain (*p* = 0.032 and *p* = < 0.001 respectively), the controls reported lower overall pain at year 7.

The patients with remaining pain at year 7 reported the greatest number of body regions with pain outlined on the pain drawing (Table 1), and significantly more painful regions than the patients with decreased pain (*p* = 0.006), the patients with low pain (*p* = 0.002), and the controls (*p* = <0.001).

### Characteristics of the patients as grouped by pain and the controls

Characteristics of the patients and the controls are presented in Table 2.

**Table 2:** Characteristics of patients with SLE as grouped by SLE-related pain and controls at year 0 and year 7

	Low pain, *n* = 50			Decreased pain, *n* = 7			Remaining pain, *n* = 7			Controls, *n* = 68		
	Year 0	Year 7	*p* ^a^	Year 0	Year 7	*p* ^a^	Year 0	Year 7	*p* ^a^	Year 0	Year 7	*p* ^a^
Age, years^b^	45 (32 to 57)	53 (38 to 64)	–	41 (36 to 52)	48 (43 to 59)	–	48 (30 to 60)	55 (38 to 67)	–	49 (39 to 59)	56 (47 to 66)	–
Sex, female/men, *n*	42/8	42/8	–	6/1	6/1	–	7/0	7/0	–	58/10	58/10	–
SLAM^b^	5 (4 to 8)	4 (2 to 7)	0.007*	12 (7 to 19)	5 (3 to 6)	0.018*	9 (5 to 14)	8 (6 to 11)	0.40	–	–	–
Change^c^ in SLAM^b^	–	1 (− 1 to 3)	–	–	9 (1 to 14)	–	–	1 (− 3 to 6)	–	–	–	–
Physicians’ reported disease activity. VAS/SLAM, mm^b^	–	5 (0 to 12)	–	–	5 (2 to 10)	–	–	15 (9 to 17)	–	–	–	–
Patients’ reported disease activity. VAS/SLAM, mm^b^	12 (8 to 22)	12 (2 to 22)	0.19	62 (49 to 70)	6 (3 to 34)	0.043*	50 (44 to 81)	50 (49 to 73)	0.69	–	–	–
Change^c^ in patient´ VAS/SLAM, mm^b^	–	5 (− 6 to 13)	–	–	56 (4 to 64)			0 (− 17 to 27)	–	–	–	–
Difference between patients’ and physicians’ global disease activity, VAS/SLAM, mm^b^	–	6 (1 to 13)	–	–	4 (1 to 24)	–	–	35 (33 to 46)	–	–	–	–
SLEDAI^b^	1 (0 to 4)	1 (0 to 4)	0.28	7 (3 to 16)	0 (0 to 2)	0.046*	2 (0 to 4)	0 (0 to 2)	0.36	–	–	–
Change^c^ in SLEDAI	–	0 (0 to 2)	–	–	7 (0 to 16)	–	–	0 (0 to 4)	–	–	–	–
SLICC^b^	0 (0 to 1)	2 (0 to 3)	< 0.001*	1 (0 to 4)	2 (1 to 4)	0.11	0 (0 to 2)	1 (0 to 3)	0.043*	–	–	–
Change^c^ in SLICC^b^	–	− 1 (− 2 to − 0)	–	–	0 (− 1 to 0)	–	–	− 1 (− 1 to 0)	–	–	–	–
Disease duration, years^b^	10 (5 to 17)	17 (12 to 24)	–	4 (1 to 11)	11 (8 to 18)	–	6 (3 to 10)	13 (10 to 18)	–	–	–	–
Treatment with glucocorticoids^d^	32 (64)	27 (54)	–	5 (71)	4 (57)	–	5 (71)	3 (43)	–	–	–	–
Current dose gluco-corticoid, po, mg^b^	3.4 (0 to 5)	2.5 (0 to 5)	0.75	7.5 (0 to 17.5)	2.5 (0 to 5)	0.09	5 (0 to 12.5)	0 (0 to 5)	0.22	–	–	–
Change^d^ in current dose gluco-corticoid, po, mg^b^	–	0 (− 1.3 to 2.5)	–	–	5 (0 to 12.5)	–	–	0 (− 2.5 to 10)	–	–	–	–
Current treatment with DMARD^d^	45 (90)	38 (76)	–	6 (86)	4 (57)	–	7 (100)	6 (86)	–	–	–	–

*p* value defines differences between inclusion (year 0) and follow-up (year 7), – not applicable or not assessed at year 0

^a^Wilcoxon matched pairs test

^b^Medians with IQR (interquartile range)

^c^Change between year 0 and year 7

^d^Numbers (%)

*Statistically significant value

There were no significant differences in age or disease duration at year 7 between the patients by pain groups in this study (Table 2).

#### Disease activity and damage in the patients as grouped by pain

At year 7, the disease activity index SLAM had decreased for the patients with low pain and for the patients with decreased pain. The patients with decreased pain had the greatest improvement between year 0 and year 7 regarding SLAM and self-reported disease activity (Table 2). However, the SLAM score remained higher for the patients with remaining pain and was significantly higher at year 7 compared to the patients with decreased pain (*p* = 0.017) as well as the patients with low pain (*p* = 0.006). Also, the physician’s estimated global disease activity on VAS/SLAM at year 7 was highest for the patients with remaining pain (Table 2) and significantly higher compared to the patients with low pain (*p* = 0.007).

The physician reported significantly lower global disease activity on VAS/SLAM at year 7 compared to the patients with low pain (*p* = < 0.001), the patients with decreased pain (*p* = 0.043), and the patients with remaining pain (*p* = 0.028). The difference between the physician’s and the patient’s estimated global disease activity was greatest for the patients with remaining pain (Table 2).

No statistical differences in SLEDAI at year 7 were found between the pain sub-groups. SLEDAI at year 7 decreased significantly only for the patients with decreased pain (Table 2). However, at year 0, the patients with decreased pain had the highest SLEDAI compared to the patients with low pain (*p* = 0.014) and to the patients with remaining pain (*p* = 0.073). Yet, there was no significant difference in SLEDAI at year 0 between the patients with low pain and those with remaining pain (*p* = 0.99).

The damage index SLICC at year 7 was highest in the patients with decreased pain (Table 2) but was not significantly higher compared to the patients with low and remaining pain.

#### Medication of the patients as grouped by pain

The use of glucocorticoids and disease modifying antirheumatic drugs (DMARDs) as well as the current dose of glucocorticoids decreased for all patients at year 7 (Table 2). The greatest reduction from year 0 to year 7 in the current dose of glucocorticoids was found for the patients with decreased pain. All patients with remaining pain used analgesics, and all but one of them used analgesics regularly (Table 1).

### Pain and pain characteristics of the patients as grouped by SLE-related pain

**Fig. 2:**
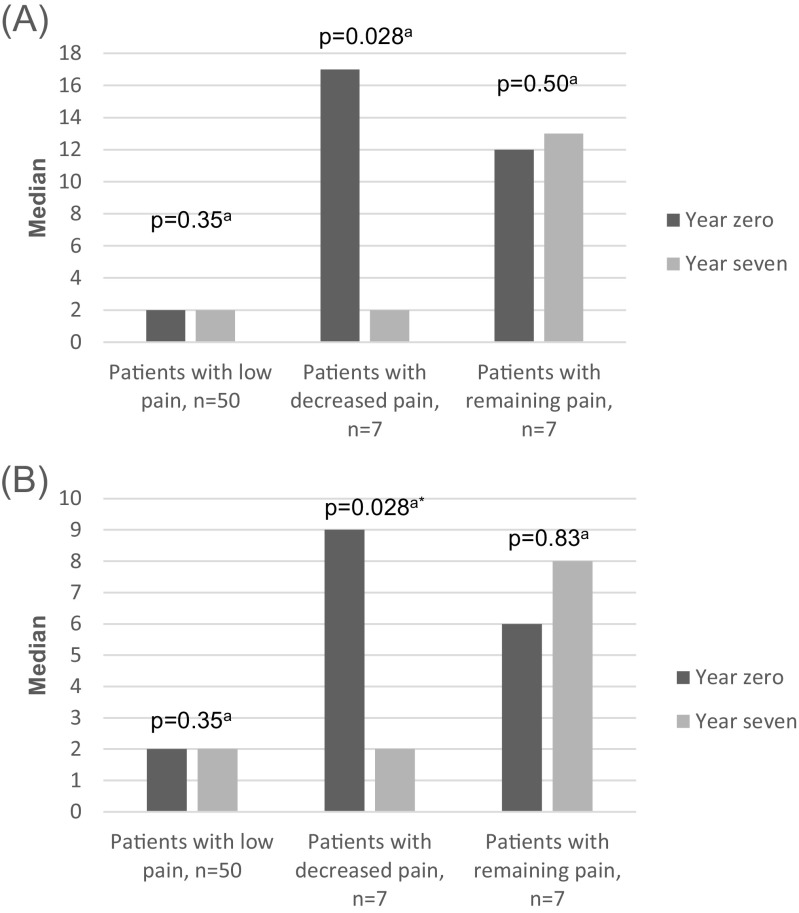
**a**, **b** SF-McGill total index and number of pain describing words by patients with SLE as grouped by SLE-related pain at year 0 and year 7. **a** SF-McGill total index (0–45). **b** SF-McGill number of descriptive words (0–15). ^a^Wilcoxon matched pairs test, *p* value defines changes between inclusion (year 0) and follow-up (year 7), *statistically significant value

According to the SF-McGill pain questionnaire, the patients with decreased pain reported a significantly lower total index as well as fewer words to describe pain and pain intensity at year 7 (Fig. 2a, b). For the patients with low and remaining pain, no significant change in pain was reported in the SF-McGill questionnaire (Fig. 2a, b).

### Fatigue of the patients as grouped by SLE-related pain and the controls

**Fig. 3:**
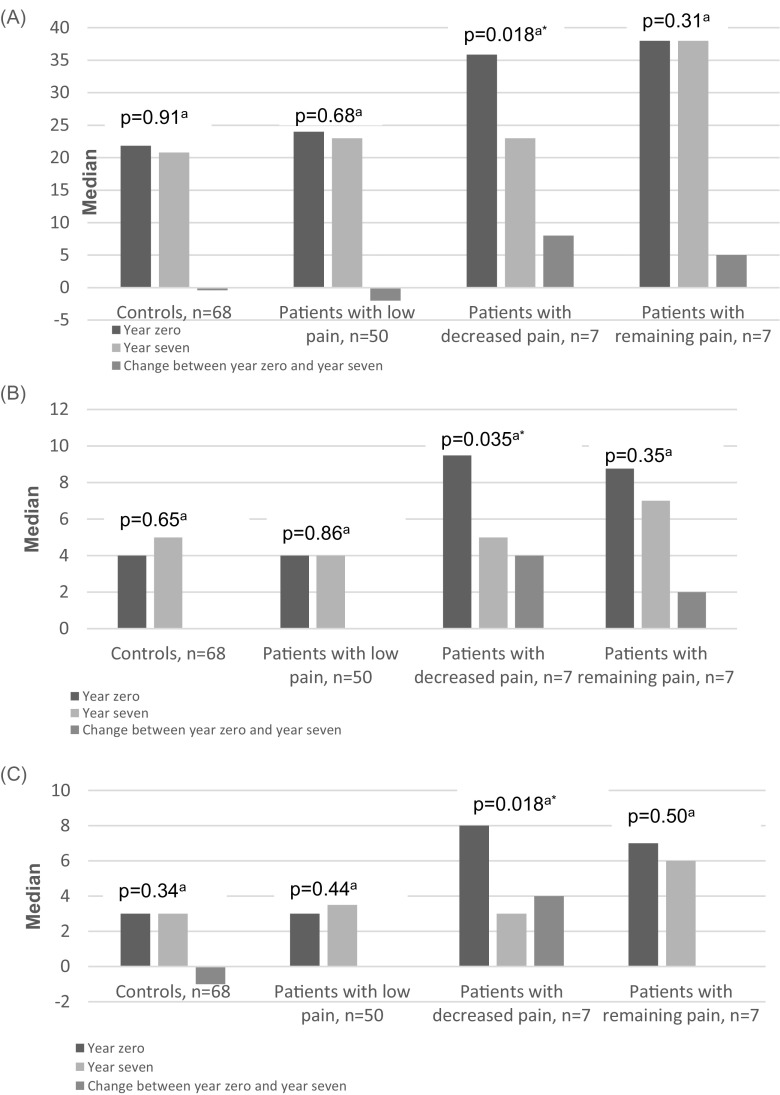
**a–c** General fatigue index, anxiety, and depression by patients with SLE grouped by SLE-related pain and controls at year 0 and year 7. **a** General fatigue index/MAF (1–50). **b** Anxiety total index (HADS) (0–21). **c** Depression total index (HADS) (0–21). ^a^Wilcoxon matched pairs test, *p* value defines differences between inclusion (year 0) and follow-up (year 7), *statistically significant value

Only the patients with decreased pain reported a significantly lower general fatigue index at year 7 compared to year 0 (Fig. 3a).

The patients with remaining pain at year 7 still experienced a significantly higher degree of fatigue than the patients with low pain (*p* = 0.017) and the patients with decreased pain (*p* = 0.017), as well as the controls (*p* = 0.001) (Fig. 3a). There were no significant differences at year 7 in reported fatigue between the patients with low pain, decreased pain, and the controls.

### Anxiety and depression of the patients as grouped by SLE-related pain and the controls

As assessed by the HADS, no symptoms of clinically significant anxiety or depression were found in any of the groups at year 7. Only the patients with decreased pain reported a significantly lower index for anxiety and depression at year 7 compared to year 0 (Fig. 3b, c). There was no significant difference between the patients as grouped by pain or the controls in the anxiety total index at year 7 (data not shown).

The patients with remaining pain had a significantly higher total index for depression at year 7 compared to the patients with decreased pain (*p* = 0.011), the patients with low pain (*p* = 0.011) and to the controls (*p* = 0.004) (Fig. 3c).

### Health-related quality of life of the patients as grouped by SLE-related pain and the controls

**Fig. 4:**
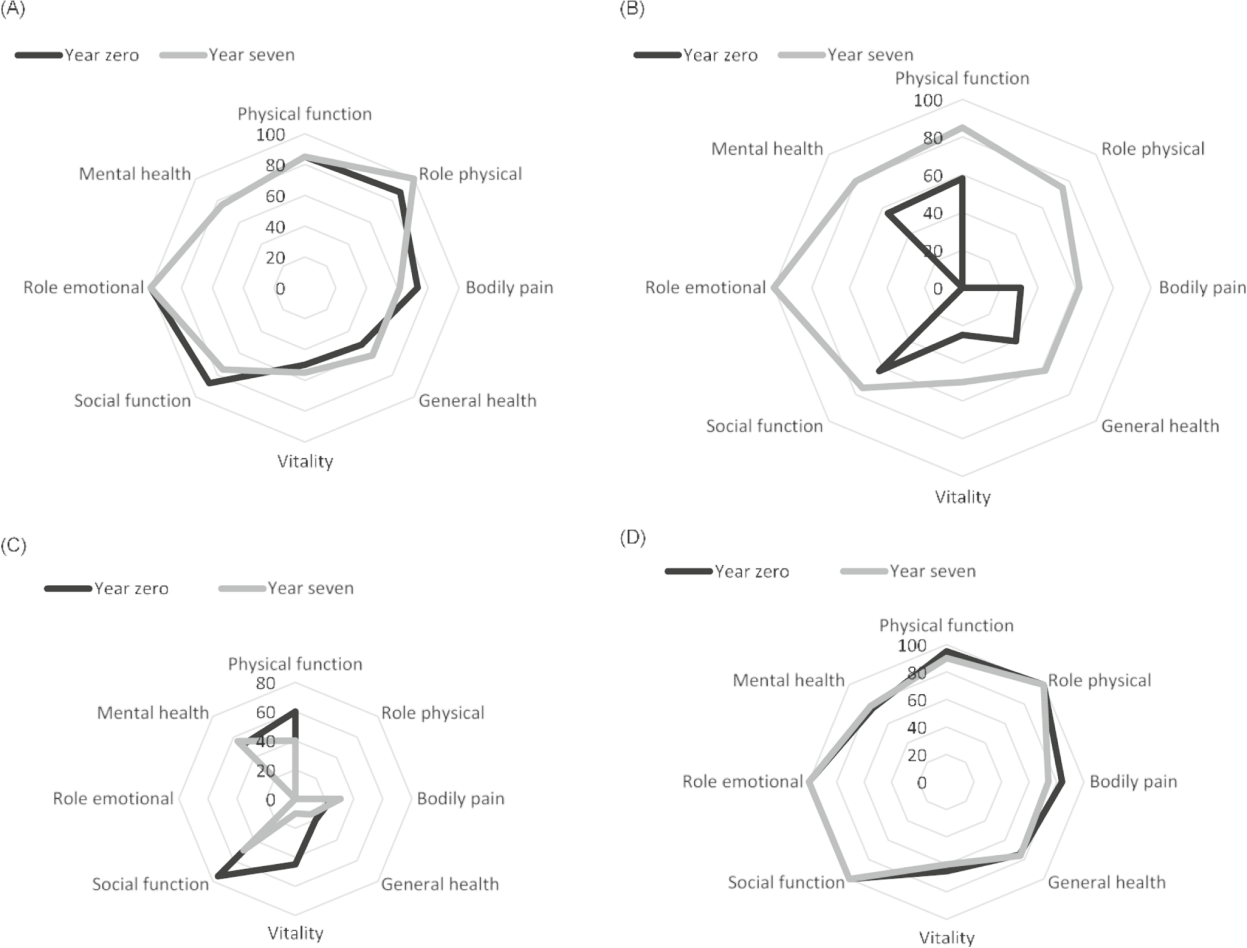
**a**–**d** Health-related quality of life (SF-36), presented in medians, by patients with SLE as grouped by SLE-related pain and controls at year 0 and year 7. **a** Health-related quality of life (SF-36) by patients with low pain (0–100). **b** Health-related quality of life (SF-36) by patients with decreased pain (0–100). **c** Health-related quality of life (SF-36) by patients with remaining pain (0–100). **d** Health-related quality of life (SF-36) by controls (0–100)

For the patients with decreased pain, there were improvements in all the dimensions of SF-36 at year 7 compared to year 0 (Fig. 4b).

In contrast, the patients with remaining pain did not report any changes in SF-36 dimensions at year 7 except for worsening in the dimension of vitality, median change (IQR), − 20 (− 35 to − 15) (Fig. 4c).

At year 7, the patients with remaining pain, compared with the patients in the decreased and low pain groups, had significantly poorer HRQoL in all dimensions of SF-36 at year 7, but not for the dimension mental health (*p* = < 0.001–0.012)*.*

There were no differences in any dimension of SF-36 at year 7 between the patients in the decreased pain and low pain groups (data not shown).

For the patients with low pain, most dimensions in SF-36 did not change from year 0 to year 7, with the exception for worsening in the dimension of *physical function*, median change (IQR) 0 (− 10 to − 5), and *bodily pain* median change 0 (IQR) (− 21 to 1) (Fig. 4a).

Among the controls, there were no significant differences in any of the dimensions in SF-36 between year 0 and year 7 (Fig. 4d). At year 7, the controls reported better health status than the patients with the remaining pain in all dimensions of SF-36 (except for mental health) (*p* = 0.005 to < 0.001). Also, the controls reported better health status than the patients with low pain in the dimensions of *physical function* (*p* = 0.016), *general health* (*p* = 0.007, *social function* (*p* = 0.023), and *bodily pain* (*p* = 0.053). At year 7, no differences were found between the controls and the patients with decreased pain.

## Discussion

In general, previous studies have shown impaired HRQoL and mental health as well as a high degree of fatigue in patients with SLE [32]. In addition to confirming our previous results, this study suggests a different pattern of symptom burden in sub-groups of patients with SLE determined by level of pain [17]. This study gives prominence to the close relationship between pain and fatigue shown by the patients with remaining pain who were the only ones who differed from the general population controls in term of fatigue. In line with our results, a previous clinical trial study reported that patients who described improvement in pain also reported improvements of HRQoL measures (SF-36), whereas patients without improvement in pain did not show any improvement in HRQoL [33].

Even if the patients with remaining pain reported higher indices in HADS and were thereby distinguished from the other participants, SLE and pain did not seem to affect the mental dimension of health as much as the physical dimension among the patients in this study. Mental health was the only dimension of SF-36 in which none of the groups differed from each other after 7 years. These results conflict with those of a previous study where SLE generally is reported to be associated with poor mental health, i.e. depression and anxiety [34]. Yet in line with our results, conflicting results between studies were also observed in a previous review [8], where the prevalence of psychiatric symptoms in patients with SLE differed between studies and where only a minority of the studies included a matched control group.

The disparity between disease activity indices as well as the discrepancy between estimations of disease activity of the physician and the patients, especially in the patients with remaining pain, assumes a risk of communication barriers. The discrepancies found in this study are in accordance with results from previous studies in which patients with SLE associated their assessment of disease activity with more subjective experiences, including the impact of the disease on life as a whole. Healthcare providers, on the other hand, base their assessments on more objective measures such as laboratory findings and physical examinations [32, 35, 36]. Yen et al. (2003) [37] found that bodily pain, by SF-36, was the most important variable for predicting clinically relevant discordance between the patient and the physician.

Higher SLEDAI at year 0 in the patients with decreased pain, in combination with a higher damage index SLICC at year 7, may suggest a higher degree of inflammatory activity at year 0 for the patients with decreased pain. In this group, after treatment and the natural course of the disease, the inflammatory activity had decreased at follow-up, yet patients had permanent damage. Despite permanent disease damage, the patients with decreased pain reported less pain and other subjective symptoms. For the patients with remaining pain, this discrepancy may illustrate the patient’s perception that pain is related to SLE and not to comorbidity, for example chronic widespread pain. For patients, it may be difficult to distinguish between inflammatory pain and other types of pain. For physicians, the symptoms of long-standing pain may be confused with SLE-related symptoms. Different types of pain require different treatments, and it is thus a major challenge for health care providers to distinguish SLE-related pain from pain of non-inflammatory origin [38, 39]. Likewise, to find out the underlying cause of pain is important in order to prevent over and under treatment of pain per se and SLE disease [3], and also to give an appropriate explanation for the cause of pain for the patient. We suggest therefore that the occurrence of long-standing widespread pain in patients with SLE should be accurately evaluated at follow-up assessments and included in prospective studies.

One limitation of this study is the relatively low number of participants in the sub-groups. However, the objective of the study was to investigate the variation over time of pain and important patient-reported outcomes in the original cohort, and thus recruitment of other patients with SLE outside this cohort was not appropriate. A strength of the study is that it had a sufficiently long follow-up to assess variation in pain and quality of life measures, and that it involved a comprehensive survey of both patients and controls, consistent with the measures used at inclusion of participants in the original study.

Taken together, the results from this 7-year follow-up study showed consistently that decreased disease-related pain is related to decreased fatigue, anxiety, and depression as well as improved HRQoL. Vice versa, the patients with remaining high levels of pain at follow-up maintained a high degree of fatigue and poor health-related quality of life. Following the principle of shared decision-making with the patient as an active participant of care, our results stress the importance of accurate monitoring of pain using self-report questionnaires incorporated in the assessment of disease burden in patients with SLE.
